# Number of Meanings and Number of Senses: An ERP Study of Sublexical Ambiguities in Reading Chinese Disyllabic Compounds

**DOI:** 10.3389/fpsyg.2018.00324

**Published:** 2018-03-29

**Authors:** Hsu-Wen Huang, Chia-Ying Lee

**Affiliations:** ^1^Department of Linguistics and Translation, City University of Hong Kong, Kowloon Tong, Hong Kong; ^2^Institute of Linguistics, Academia Sinica, Taipei, Taiwan; ^3^Institute of Neuroscience, National Yang-Ming University, Taipei, Taiwan; ^4^Institute of Cognitive Neuroscience, National Central University, Taoyuan, Taiwan; ^5^Research Center for Mind, Brain, and Learning, National Chengchi University, Taipei, Taiwan

**Keywords:** Chinese compounds, sublexical semantic ambiguity, ERPs, N400, homonymy, polysemy

## Abstract

In English, an extensive body of work in both behavioral and neuropsychological domains has produced strong evidence that homonymy (words with many distinct meanings) and polysemy (many related senses) are represented, retrieved, and processed differently in the human brain. In Chinese, most words are compounds, and the constituent characters within a compound word can have different meanings and/or related senses on their own. Thus, in order to resolve lexical ambiguity in Chinese, one has to consider the composition of constituent characters, as well as how they contribute to whole word reading, known as “sublexical ambiguity.” This study investigates how two types of sublexical ambiguity affect Chinese word processing. The number of meanings (NOM) and the number of senses (NOS) corresponding to the first character of Chinese compounds were manipulated in a lexical decision task. The interactions between NOM and NOS were observed in both behavioral results and N400s, in which NOM disadvantage effect was found for words with few-senses only. On the other hand, the NOS facilitation effect was significant for words with multiple-meanings (NOM > 1) only. The sublexical ambiguity disadvantage suggested that semantically unrelated morphemes are represented as separate entries. For characters with multiple meanings, one orthographic form is associated with more than one morphemic representation. In contrast, the sublexical sense advantage supported the idea that semantically related senses that shared a morphological root are represented within a single entry. The more senses listed in a morphological root, the stronger representation will be formed. These results suggest that two types of sublexical ambiguities are represented and processed differently in Chinese word recognition models and also demonstrate that how they interact with each other in the mental lexicon.

## Introduction

“Words and meanings do not always form one-to-one correspondences.” The majority of words are, in fact, extensively associated with multiple meanings — which has been referred to as lexical ambiguity. For lexical items, two different types of ambiguity have been distinguished. Homonymous words, such as *bark*, have two (or more) semantically unrelated meanings associated with a single word form. The word *bark* can refer either to the sound made by a dog, or to a part of a tree. Polysemous words, on the other hand, such as *paper*, have two or more semantically related senses associated with one word form. *Paper* can refer to a material, as in “making something out of paper,” or to the content of a publication, as in “He is reading an interesting paper.”

In the visual word recognition literature, studies have reported that words with multiple meanings yield faster response times than words with few meanings, when all words are matched for frequency (Rubenstein et al., 1970; Jastrzembski, 1981; Kellas et al., 1988; Millis and Button, 1989; Borowsky and Masson, 1996; Hino and Lupker, 1996; Azuma and Van Orden, 1997; Lichacz et al., 1999), the so-called “ambiguity advantage effect.” Typically, these results were explained by assuming that the processing benefit for ambiguous words come from having multiple entries in the lexicon. However, this assumption has been challenged more recently, as ambiguity can arise in different ways. Despite pervasive reports of ambiguity advantages in the literature, only few studies explicitly dissociated ambiguity between unrelated meanings (homonymy) and ambiguity between related senses (polysemy). Indeed, most studies have often used the two terms interchangeably (see Klein and Murphy, 2001, for a discussion of this issue).

To further distinguish the effects of having multiple unrelated meanings (homonymous words) from the effects of having multiple related senses (polysemous words), Rodd et al. (2002) reanalyzed both high- and low- ambiguous words used in previous studies (e.g., Millis and Button, 1989; Borowsky and Masson, 1996; Azuma and Van Orden, 1997). They found that the number of meanings (NOM) of high-ambiguous words did not differ from low-ambiguous words; instead, high-ambiguity words had a significantly higher number of related senses. The authors suggested that the ambiguity advantage effect shown in previous studies might reflect an advantage for polysemous words with many related senses. In addition, they examined two types of ambiguity in a lexical decision task and demonstrated that homonymy and polysemy produce opposite effects. While there was a processing disadvantage for words that had multiple unrelated meanings (homonyms, e.g., *bank*), there was a processing advantage for words that had many interrelated senses (polysemes, e.g., *paper*). These effects of ambiguity disadvantage and sense advantage have been replicated in several other studies (Frazier and Rayner, 1990; Klepousniotou, 2002; Klepousniotou et al., 2012) and are indicative of representational and processing differences between homonymy and polysemy.

In addition to the lexical decision results, many studies have attempted to understand the online brain responses as people read words by using electroencephalography (EEG) or magnetoencephalography (MEG) methods (Klepousniotou et al., 2012). Converging evidence from MEG has shown that the peak latency of the M350 component which is sensitive to lexical activation was modulated by lexical ambiguities. Homonyms showed later M350 latency than non-homonyms. In contrast, words with many senses showed earlier peak latency of M350 than words with few senses (Beretta et al., 2005; Pylkkänen et al., 2006). The opposite patterns of M350 latency observed between homonymy and polysemy suggest differential neurocognitive representations for the two types of ambiguity. For a homonymous word, the orthographic code is associated with multiple semantic representations. The one-to-many mapping from form to meaning delays the recognition of a homonymous word (Beretta et al., 2005). On the other hand, related senses of a polysemous word were stored as a single core meaning. Words with many senses are semantically richer, and thus, easier to recognize than words with fewer senses (Rodd et al., 2002).

The above mentioned research is mainly carried out by using alphabetic writing system such as English. The question that arises, however, is whether distinctions between homonymy and polysemy in English are generalizable to Chinese, a different orthographic system. The predominant word type of Chinese is disyllabic compound. In other words, the only productive morphological processing in Chinese is compounding. According to the Chinese words corpus of Academia Sinica (1998), more than 60% of the compounds have at least one orthographic neighbor that shares the same constituent character at the same position. The greater the orthographic neighborhood size of the first constituent character, the longer response time is in recognizing the word (Huang et al., 2006). More importantly, the constituent characters of compounds are physically distinguishable and can be mapped onto syllables and morphemes. And further, each constituent character can have different meanings and/or related senses in its own right. Thus, “the constituent characters of Chinese disyllabic compounds not only represent as perceptual units at the orthographic level, but also represent as semantic units at the morphemic level” (Hoosain, 1991; Huang H.W. et al., 2011). The resolution of lexical ambiguity in Chinese has to consider the nature of character compositions and how the activation of character meanings contribute to whole-word meaning, which makes the issues of homonymous and polysemous representations more complex for Chinese words. For example, the character (hua1) has at least two meanings (*flower* and *to spend*), leading to the question: how do readers choose the appropriate meaning when is used as the first constituent character in a compound such as (hua1 yuan2; *flower garden*) during reading? This issue is known as “sublexical ambiguity resolution.”

In order to examine whether semantic representations of the constituent characters were accessed in the process of Chinese word recognition, Huang H.W. et al. (2011) manipulated subjective semantic ambiguity of the first constituent character and matched the orthographic neighborhood sizes in a lexical decision task. They found that words with high sublexical semantic ambiguity elicited smaller N400s than those with low sublexical semantic ambiguity, which is inconsistent with other ambiguity studies (e.g., Beretta et al., 2005). The authors raised a possible problem with the subjective ambiguity rating itself that used to estimate the NOMs associated with a character. That is, the homonymy and polysemy distinction may not be determined by subjective ratings alone. Therefore, the current study utilized the meaning indices provided by the Chinese Wordnet, a lexical ontology database for Mandarin Chinese (Huang and Hsieh, 2010). We aimed to investigate whether multiple meanings and related senses of the first character within a compound are represented and processed differently in reading Chinese.

In Chinese Wordnet, a homonymous example such as 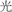 *kuang*1 has three unrelated meanings: (1) “light,” as in 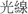 *kuang1 xian4* “streams of light”; (2) “naked,” as in 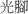 *kuang1 jiao3* “barefoot”; (3) “simply,” as in 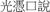 *kuang1 ping2 kou3 shuo1* “simply saying.” An example of a polysemous element having several senses is 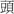 *tou2*, meaning “head,” literally as in 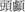 *tou2 lu2* “skull,” and with semantic extensions as in 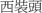 *xi1 zhuang1 tou2* “a kind of hairstyle,” 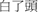 *bai2 lë tou2* “hair turning white,” and 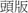 *tou2 ban3* “the front page of a newspaper,” and so on. The study of sublexical ambiguity resolution in reading Chinese words can shed light on models for Chinese compound word processing. In the current study, we manipulate two types of sublexical ambiguity, the NOM and number of senses of a meaning (NOS), both corresponding to the first character of a compound. The N400 component, an event-related potential (ERP) response associates with the lexical activation and semantic processing of words would be used to examine how NOM and NOS influence the mental representations. If each meaning of a character has a separate morphemic representation, words with an ambiguous first character should show larger N400s and longer response times than those with an unambiguous first character (sublexical ambiguity disadvantage effect). If semantically related senses are represented within a single entry, we would predict that words with many senses will elicit smaller N400s and shorter response times than those with fewer senses (sublexical sense advantage effect). Additionally, if the distinctiveness of a specific morphemic representation is determined by the number of senses (NOS), we would expect that the more senses listed in an entry, the stronger representation it will form at the morphemic level.

## Materials and Methods

### Participants

Data were obtained from 25 right-handed native Chinese speakers between the ages of 18 and 25 (mean age 22.1 years); participants received cash for their time. Participants were screened for normal vision. This study was approved by the Ethics Committee of the Institute of Linguistics, Academia Sinica. Written informed consent was obtained from all participants.

### Stimuli

Stimuli consisted of 120 Chinese disyllabic compound words which were selected from the Academia Sinica balanced corpus (Huang and Chen, 1998). The words were divided into four subsets by orthogonally manipulating the two types of sublexical properties – the NOMs corresponding to the first character (NOM, one meaning vs. multiple meanings) and the NOS of the first character (NOS, few-senses vs. many-senses). The NOMs and NOS were collected from the Academia Sinica Chinese Wordnet (Huang and Hsieh, 2010).

The two levels of the NOM variable were defined in the following way: unambiguous words had a first character with only one meaning (NOM = 1, mean = 1) whereas the ambiguous words had a first character whose NOMs varied from 2 to 7 (NOM > 1, mean = 3.1). The two levels of the NOS variable were defined as such: few-senses words had a first character with between 1 and 3 senses (mean = 2.3), whereas many-senses words had a first character whose NOS varied from 6 to 23 (mean = 11.6). It is important to point out that there are two values for the NOS a word has: one refers to sense corresponding to the target word meaning, and the other refers to total senses that a given character has, across all meanings. Take an ambiguous character *kuang*1 as an example, which has three different meanings (*light, naked*, and *simply*): there are four senses for the meaning of *light*, three senses for the meaning of *naked*, and one sense for the meaning of *simply*. Across all three of its meanings, there are eight senses for the character *kuang*1. In our stimuli, senses corresponding to the target meaning of the word were manipulated. The total numbers of senses were matched in three conditions: one meaning, many-senses words (NOM = 1, many-senses), multiple meanings, few-senses words (NOM > 1, few-senses), and multiple meanings, many-senses words (NOM > 1, many-senses). It is impossible to match the total NOS for NOM = 1, few-senses condition. Other possible confounding factors such as word frequency (WF), neighborhood size of the first character (NS1), neighborhood size of the second character (NS2), and the NOMs corresponding to the second character were controlled (see **Table 1**). For the lexical decision task, a list of 120 pseudowords was generated as NO trials. Pseudowords were constructed from concatenations of two characters that do not occur in the word corpus. And pronunciation of pseudoword was controlled not to resemble the pronunciations of the real words. In total, each participant saw 240 trials.

**Table 1:** Descriptive statistics for the stimuli.

	WF	NS1	NS2	NOM	NOS
NOM = 1, few-senses	16.7	21.7	16.3	1.0	2.0
NOM = 1, many-senses	15.4	22.2	16.0	1.0	12.9
NOM > 1, few-senses	15.9	22.7	22.6	3.1	2.5
NOM > 1, many-senses	16.5	24.8	20.5	3.0	12.2

### Procedure

Participants viewed the stimuli sitting 70 cm in front of a monitor in a sound-proof room. They were instructed to read the words for comprehension and to respond to the word as quickly and accurately as possible with a lexical decision judgment via button press. They pressed the left mouse button if the stimulus was a real Chinese word or ‘no’ with the right mouse button if the stimulus was a pseudoword. A 20-trial practice with 10 words and 10 pseudowords familiarized subjects with the task. At the start of each trial, a white cross appeared centrally for 500 ms. Next, the stimulus was presented for 500 ms, followed by a blank screen for a maximum of 1300 ms or until the participant made a judgment. Participants were encouraged to minimize blinks or eye movements during this period. At the end of each trial, a capital B was displayed for 1500 ms indicating that blinking was now allowed. The inter-trial interval was 1500 ms. There were four blocks of trials, with 60 trials per block. Between blocks, participants took a short break.

### EEG Recording and Processing

The electroencephalograms (EEG) was recorded from 64 sintered Ag/AgCl electrodes mounted on a cap (QuickCap, Neuromedical Supplies, Sterling, United States). Eye movements were monitored via electrodes placed on the outer canthus of each eye. Blinks were detected by a pair of electrodes placed on the supraorbital and infraorbital ridges of the left eye. Electrode impedances were kept below 5 kΩ. Signals were amplified by SYNAMPS2^®^ (Neuroscan, Inc.) with a 0.1–100 Hz bandpass and digitized at 500 Hz. Data were referenced to the average of left and right mastoids.

ERPs were computed from 100 ms prestimulus baseline to 922 ms poststimulus onset. Epochs contaminated by eye movements, blinks or muscle activities were rejected offline. A band-pass filter of 0.01–30 Hz (zero phase shift mode, 12 dB/oct) was employed. ERPs were calculated for each subject and condition for correct trials only. Statistical analyses were performed on mean amplitudes in the N400 and late positive complex (LPC) time windows after Greenhouse–Geisser correction.

## Results

### Behavior

Reaction time and accuracy data (see **Table 2**) were subjected to repeated-measures analyses of variance (ANOVAs) for words with two levels of NOMs and two levels of NOS as within-subject factors. Incorrect responses or reaction times that exceeded 2 SD from the subject’s mean were removed from the analysis.

**Table 2:** Mean reaction times and accuracy.

	RT (ms)		Accuracy (%)	
	NOS few-senses	NOS many-senses	NOS few-senses	NOS many-senses
NOM = 1	637.0 (74.1)	613.6 (83.3)	97.3 (0.03)	97.9 (0.04)
NOM > 1	659.9 (81.7)	608.3 (71.4)	94.1 (0.04)	95.7 (0.03)

For reaction times, the NOM main effect was marginally significant in the participants analysis [*F*_1_(1,24) = 3, *p* = 0.09], the differences in the items analysis was not significant [*F*_2_(1,29) = 0.4], but the result showed the same trend as in the participants analysis, such that words with multiple meanings were responded to slower than words with one meaning. The NOS main effect was significant in the participants analysis [*F*_1_(1,24) = 28.5, *p* < 0.001] and also in the items analysis [*F*_2_(1,29) = 18, *p* < 0.001]: words with many senses were responded faster than those with few senses. The interaction between NOM and NOS was also significant in both participants analysis [*F*(1,24) = 13.3, *p* < 0.01] and items analysis [*F*(1,29) = 7.1, *p* < 0.01]. *Post hoc* analysis showed that the facilitative effects of NOS were significant for both words with one meaning [*F*(1,48) = 8.5, *p* < 0.01] and words with multiple meanings [*F*(1,48) = 41.4, *p* < 0.001] in the participants analysis. In the items analysis, facilitative effects of NOS was only significant for words with multiple meanings [*F*(1,56) = 21.7, *p* < 0.001]. However, NOM showed significant inhibitory effects on RTs only for words with few-senses [*F*(1,48) = 12.9, *p* < 0.01] in the participants analysis and the same trend in the item analysis [*F*(1,56) = 3, *p* = 0.09], but there was no NOM effect for words with many-senses in either the participants analysis or item analysis (*F*s < 1).

For accuracy, the NOM main effect was significant [*F*_1_(1,24) = 18.4, *p* < 0.01]; words with multiple meanings were responded to less accurately than words with one meaning. The NOS main effect was also marginally significant [*F*_1_(1,24) = 3.4, *p* = 0.09]: words with many-senses were responded more accurately than words with few-senses. The interaction between NOM and NOS did not reach significance [*F*_1_ < 1]. No effect reached significance in the item analysis.

### ERPs

**FIGURE 1:**
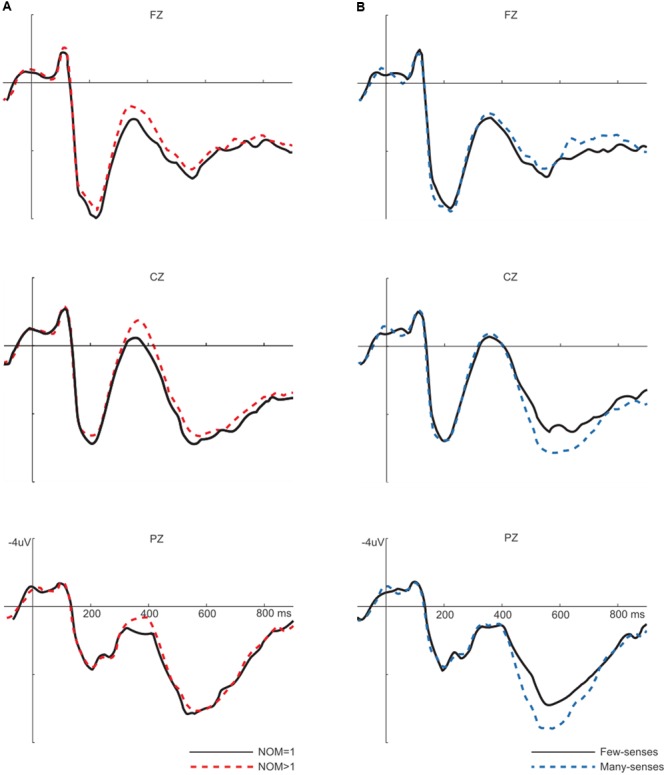
Left **(A)** sublexical ambiguity (NOM) main effect. Words with multiple meanings (NOM > 1) elicited more negative N400s than words with one meaning (NOM = 1). Right **(B)** sublexical sense (NOS) main effect. Words with many-senses showed a more positive LPC than words with few-senses.

**Figure 1** overlays the grand average ERPs at three representative channels to words with multiple meanings vs. one meaning (**Figure 1A**) and words with many-senses vs. few-senses (**Figure 1B**). All conditions elicited typical brain responses for visual stimulation, including the posterior P1, N1, and P2, and the anterior N1 and P2. Following the sensory components, all conditions elicited a negative-going wave (N400), and then a late positive component (LPC). Sublexical ambiguity effects were analyzed for the N400 (250–450 ms), and LPC (450–650 ms) using ANOVAs with NOM (NOM = 1 vs. NOM > 1), number of word sense (few-senses vs. many-senses), and electrodes in regions of interest. For each ANOVA, the Greenhouse–Geisser adjustment to the degrees of freedom was applied to correct for violations of sphericity associated with repeated measures. For all *F* tests with more than one degree of freedom in the numerator, the corrected *p*-value is reported. The analysis of N400 and LPC were conducted separately on the data derived from midline and lateral sites. In the midline analysis, factors of NOM, NOS, and electrode (FZ, FCZ, CZ, CPZ, and PZ) were included as within-subject factors. As for the lateral analysis, factors of NOM, NOS, laterality (left and right), and electrode (F3/4, FC3/4, C3/4, CP3/4, and P3/4) were used as within-subject factors.

#### N400

**FIGURE 2:**
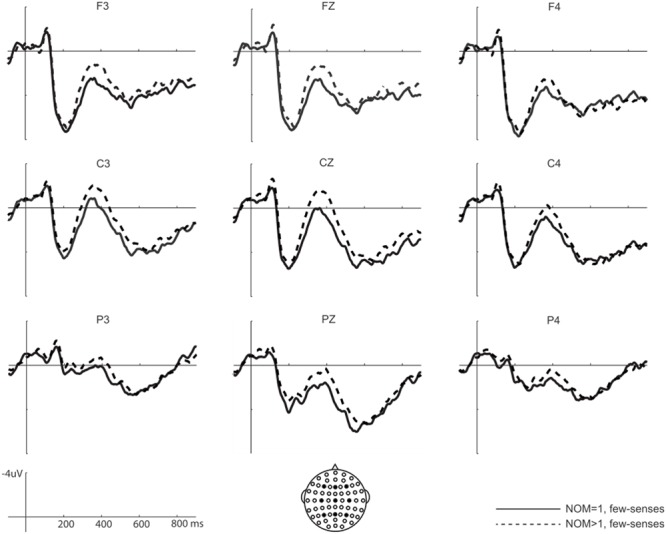
Sublexical ambiguity effect for words with few-senses on N400. Words with multiple meanings (NOM > 1, few-senses) elicited more negative N400s than words with one meaning (NOM = 1, few-senses).

**FIGURE 3:**
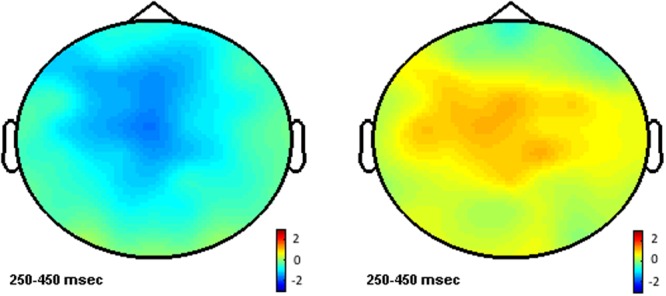
**Left**: topographic map for the NOM effect for words with few-senses. **Right**: topographic map for the NOS effect for words with multiple meanings.

**FIGURE 4:**
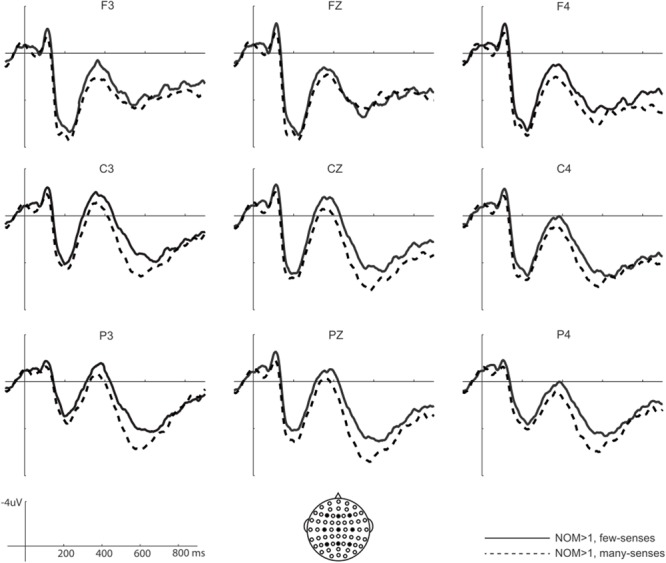
Sublexical sense effect for words with multiple meanings on N400. Words with many-senses (NOM > 1, many-senses) elicited less negative N400s than words with few-senses (NOM > 1, few-senses).

Neither NOM nor NOS main effects reached significance on this time window (all *F*s > 1). Significant interactions between NOM and NOS were found in both midline and lateral analyses [midline: *F*(1,24) = 8.2, *p* < 0.01; lateral: *F*(1,24) = 6.4, *p* < 0.05]. *Post hoc* comparisons showed that NOM effect was only significant for words with few-senses [midline: *F*(1,24) = 9.4, *p* < 0.01; lateral *F*(1,24) = 7.3, *p* < 0.01], but not for words with many-senses (*F*s < 1). As can be seen in **Figures 2, 3**, words with multiple meanings (NOM > 1, few-senses) elicited more negative N400s than those with only one meaning (NOM = 1, few-senses). In contrast, the NOS effect was significant for words with multiple meanings (NOM > 1) [midline: *F*(1,24) = 4.3, *p* < 0.05; lateral *F*(1,24) = 4.5, *p* < 0.05], in that words with many-senses showed a less negative N400 than words with few-senses (**Figures 3, 4**). The NOS effect was not significant for words with one meaning (*F*s = 1). No other effect reached significance in this time window.

#### LPC

Number of meanings main effects were not significant in this time window (*F*s < 1). NOS main effects were significant on the LPC [midline: *F*(1,24) = 8.2, *p* < 0.05, lateral: *F*(1,24) = 13.4, *p* < 0.05], with more positive responses to words with many-senses than those with few-senses. The interaction between NOS and electrode was significant [midline: *F*(2.3,54.6) = 15.6, *p* < 0.01; lateral: *F*(3,71.6) = 7.8, *p* < 0.01]. *Post hoc* comparisons showed that the sense effect was significant at central to posterior sites (CZ, CPZ, PZ, C3/4, CP3/4, and P3/4, *p* < 0.0001). No other effect reached significance in this time window (**Figure 1B**).

## Discussion

In this study, we demonstrated that sublexical ambiguity influences the recognition of Chinese words by showing that both the NOMs and the NOS of constituent characters in a disyllabic compound impact ERPs and behavioral responses to these words. This suggests that readers access semantic representations of a disyllabic compound through the orthographic and semantic representations of constituent characters. More importantly, two opposite effects of sublexical ambiguity were characterized on the lexical decision performance. One is the sublexical ambiguity disadvantage, in which words with multiple meanings at the sublexical level *delay* the word recognition relative to words with one meaning. The other effect is the sublexical sense advantage effect, in which words with many senses at the sublexical level *facilitate* word recognition relative to words with few senses. The sublexical ambiguity disadvantage and the sublexical senses advantage effects on both behavioral responses and ERPs are consistent with prior research for the ambiguity effects at the lexical level in English (e.g., Rodd et al., 2002, 2004; Beretta et al., 2005; Klepousniotou et al., 2012) (specifically, in showing both a disadvantage for words with multiple meanings, and an advantage for words with multiple senses).

Our behavioral findings of sublexical ambiguity disadvantage suggested that the mapping between orthographic form and morphemic representation is one-to-many for characters with multiple meanings. Semantically unrelated morphemes are represented as separate entries. For unambiguous words that had a first character with only one meaning, the mapping between orthography and morpheme is a straightforward one-to-one mapping. When retrieve the meaning of a sublexically ambiguous word, a competitive process occurs between multiple meanings. And thus, it takes longer time to select the appropriate meaning for words had a first character with multiple meanings. The N400 amplitudes mirror behavioral findings in showing that words with ambiguous first character elicit larger N400s than words with unambiguous first character. On the other hand, the sublexical sense advantage suggested that semantically related senses share a morphological representation within a single entry, and the NOS modulates the clarity of the morpheme. When a character has many-senses, the meaning is used in a wider range of contexts, thus, a stronger morphemic representation is formed. As the results, the stronger representation for characters with many-senses facilitates the recognition of its morphological root than those with few-senses. This interpretation is consistent with the hypothesis of “sense-related effects” (Pylkkänen et al., 2006; Huang C.Y. et al., 2011).

We also demonstrated interactive effects for the factors of NOM and NOS. The NOM effect on the N400 amplitude is prominent for words with few-senses but not for words with many-senses, again words with multiple meanings elicited more negative N400s relative to words with one meaning. As we mentioned earlier, the related senses might modulate the clarity of a morphemic representation within an entry, the more senses listed in a morphological root, the stronger representation will be formed. Then, it would be much easier to solve the sublexical ambiguity when the morphemic root has a stronger representation. When processing sublexically ambiguous words with many-senses, the N400 amplitudes would be roughly equal in size as those unambiguous many-senses words. In contrast, it is difficult to solve the sublexical ambiguity when the morphemic root has a weak representation (few senses) among competitors. As a result, the N400 amplitudes were larger for words that are sublexically ambiguous with few-senses than those unambiguous few-senses words.

The hypothesis of sense-relatedness effect can also explain that the sense facilitation was mainly found for words had an ambiguous constituent character but not for words had a constituent character with one precise meaning. For characters with one meaning, there is no need to solve the competition among different morphological roots. Therefore, the NOS might not play a role under these circumstances. Only when a character has multiple meanings, a morpheme with many-senses will be recognized more rapidly than those with few-senses. Again, the N400 responses are consistent with the behavioral findings—the N400 amplitude is reduced for words with an ambiguous many-senses character than words with an ambiguous few-senses character.

Furthermore, words with many-senses showed more positive LPCs than words with few-senses. This component has been found due to repeated items, particularly in performing of the explicit memory task (Rugg and Doyle, 1994). The LPC indexes an explicit evaluative aspect of semantic processing. In the literature, the LPC magnitude has shown a positive correlation with memory strength (e.g., Rugg and Doyle, 1994). More positive LPCs for words with many senses may indicate an easier retrieval process of the word meaning.

Most models of lexical processing are based on monosyllabic words, like *bark* and *paper*, however, words cover a wide spectrum of morphological type and complexity, ranging from monomorphemic words to multimorphemic words. Despite wide acceptance that words are “decomposed” into their constituent morphemes when processing multimorphemic words, there is not a wide consensus on how or when this decomposition occurs (Stites et al., 2016). When modeling the processing of more complex words such as compound words, the nature of the morphological representation needs to be established. This issue is particularly important for recognition of Chinese words, because each Chinese character is a physically distinct unit that can map onto one or even multiple morphemes, and two-character compounds make up more than 80% of the Chinese words. Although the findings of Chinese character recognition by using both a character decision task and a word decision task have suggested a lemma level of morphological representation to capture the relationship between word forms and meanings (Taft, 2006), the exact nature of this morphological representation in lexical memory is still unclear.

In sum, this study investigated the morphological representations of Chinese two-character words and used NOM and NOS listed in the Chinese Wordnet as indices of two types of sublexical ambiguity. Our results support representational differences for NOM and NOS. Unrelated meanings of a morpheme are represented as separate entries; in contrast, related senses of a morpheme are represented as a single entry. Moreover, the distinctiveness of the morphemic representation for a word seems depend on the NOS – more senses within one entry, a more distinct representation is formed. These data add to the accumulating evidence suggesting that the establishment of morphemic representations between form and meaning is crucially required in reading Chinese.

## Author Contributions

H-WH collected, analyzed, and interpreted the data and wrote the paper. C-YL interpreted the data and wrote the paper.

## Conflict of Interest Statement

The authors declare that the research was conducted in the absence of any commercial or financial relationships that could be construed as a potential conflict of interest.
